# Cold-Active Lipase from the Ice Cave *Psychrobacter* SC65A.3 Strain, a Promising Biocatalyst for Silybin Acylation

**DOI:** 10.3390/molecules29215125

**Published:** 2024-10-30

**Authors:** Victoria I. Paun, Sabina G. Ion, Giulia R. Gheorghita, Iunia Podolean, Madalina Tudorache, Cristina Purcarea

**Affiliations:** 1 Department of Microbiology, Institute of Biology Bucharest of the Romanian Academy, 296 Splaiul Independentei, 060031 Bucharest, Romania; ioana.paun@ibiol.ro (V.I.P.); gheorghita.giulia@yahoo.com (G.R.G.); 2 Department of Inorganic Chemistry, Organic Chemistry, Biochemistry and Catalysis, Faculty of Chemistry, University of Bucharest, 4-12 Regina Elisabeta Blvd., 030016 Bucharest, Romania; ion.sabina.gabriela@chimie.unibuc.ro (S.G.I.); iunia.podolean@chimie.unibuc.ro (I.P.)

**Keywords:** extremozyme, cold-active lipase, ice cave bacteria, silybin acylation, biocatalyst

## Abstract

Cold-active lipase from the psychrophilic bacterial strain *Psychrobacter* SC65A.3 isolated from Scarisoara Ice Cave (Romania) was cloned and characterized as an extremophilic biocatalyst for silybin acylation. Structural analyses highlighted conserved motifs confirming a functional lipase and the presence of primary structure elements for catalysis at low temperatures. The recombinant enzyme (PSL2) heterologously expressed in *Escherichia coli* was purified in one step by affinity chromatography with a yield of 12.08 ± 1.72 µg L^−1^ of culture and a specific activity of 20.1 ± 3.2 U mg^−1^ at 25 °C. Functional characterization of PSL2 showed a neutral (7.2) optimal pH and a high thermal stability up to 90 °C. Also, this lipase was stable in the presence of different organic solvents, with 60% residual activity when using 20% DMSO. Kinetic measurements indicated performant catalytic efficiency of PSL2 for different short and long chain fatty acids, with Km in the mM range. The catalytic activity of PSL2 was assessed for silybin acylation with various fatty acids and fatty acid methyl esters, demonstrating a 90% silybin conversion when methyl decanoate ester was used. This result clearly highlights the biocatalytic capability of this new cold-active lipase.

## 1. Introduction

Lipases (glycerol ester hydrolases; EC 3.1.1.3) play crucial roles in various biochemical processes, catalyzing the breakdown of triglycerides, as well as the esterification and transesterification of water-insoluble fatty acid substrates [1,2,3]. These enzymes are structurally defined by the α/β hydrolase fold, a conserved catalytic triad (SD/EH), and a semi-conserved pentapeptide motif (GXSXG) that forms an oxyanion hole, which classifies them into 15 superfamilies and 32 homologous families [2,4,5,6]. Microbial lipases can be grouped into six distinct families (I to VI) based on amino acid sequence homology, with a large majority belonging to families I and II, while cold-active lipases are generally classified under family IV [7,8,9]. 

Lipases are key catalysts in various industries, representing about 30% of the global enzyme market [2]. They are widely used in sectors such as pharmacology [10,11], fine organic synthesis [12,13], the food industry [14,15,16], the detergent industry [11,17], biomedical applications [18], biosensors [19], and biodiesel production [11]. Their industrial value is attributed to their stability in organic solvents, versatility with substrates, high selectivity, activity at ambient temperatures, ease of production, and independence from cofactors for catalysis [4,11,13]. For the last few decades, lipase-based biocatalysis has become a highly attractive tool in industrial organic synthesis due to its broad substrate specificity, activity at low temperatures, and neutral pH [4,20]. In addition to high chemoselectivity, regioselectivity, and stereoselectivity [21,22], their tolerance to organic solvents enable the conversion of poorly soluble small molecules in aqueous environments to obtain the desired target products [22].

The most studied and industrially utilized lipases are of microbial origin, derived from bacterial and fungal species of *Pseudomonas*, *Bacillus*, *Cryptococcus*, *Burkholderia*, *Fusarium*, *Rhizomucor*, *Aspergillus*, *Geotrichum*, and *Candida* [23]. These enzymes were discovered in environments associated with food spoilage or a high oil content due to their ability to utilize fat as a carbon source [4]. Among these, the genera *Pseudomonas* and *Bacillus* are especially recognized as prime sources of lipases [24,25]. To date, several recombinant lipases from mesophilic microorganisms have been obtained and characterized as putative catalysts for a broad range of industrial applications [23,26,27]. However, despite the wide range of production sources, the balance between cost and performance remains a critical challenge for industrial applications [28]. Consequently, developing new cost-effective lipases is a major focus of current research.

More recently, extreme biocatalysis, representing a highly attractive, sustainable, cost-effective, and environmentally friendly option [29], has focused on lipases from thermophilic and hyperthermophilic microorganisms due to their enhanced thermal stability [30,31,32,33,34]. Meanwhile, few reports are available to date on the characterization of the corresponding cold-active lipases [35,36]. During the last few decades, the use of cold-active enzymes in biotechnologies raised a particular interest due to their high stability and catalytic activity at low temperatures, in addition to a variable fatty acid substrate specificity [36,37,38,39]. These extremozymes have evolved a series of structural features that confer a higher flexibility around the active site in order to function at low temperatures, which can translate to a low activation enthalpy, a low substrate affinity, and a high specific activity, while the maximum activity is found at lower temperatures [35,36,37]. Despite these characteristics, only a limited number of cold-active lipases have been commercially utilized in the medicine, synthetic chemistry, food, and detergent industries as compared to the mesophilic and thermophilic homologous enzymes [2]. 

Although lipase production is typically extracellular [24], the use of heterologous bacterial and fungal expression systems is highly valuable for generating large quantities of recombinant enzymes at low cost [40].

Cryophilic microbial species hosting cold-active enzymes were retrieved from various cold habitats [41,42,43]. Among these, ice caves represent a fairly unexplored reservoir of novel cold-adapted microbes and biomolecules [44]. Our recent investigation of bacterial communities from a 13,000 years old ice core from Scarisoara Ice Cave (Romania) [45] led to the isolation and characterization of a new psychrophilic *Psychrobacter* SC65A.3 strain from ice deposits accumulated about 5000 years ago in this cave. Extracellular fractions of this cave bacterium evidenced stable lipolytic activity, including the acylation of silybin, the main silymarin component, using methyl fatty acid esters [39]. The genome sequence of this strain was also determined [46] and is among the very limited number of genomes from psychrophiles determined to date [36]. 

The natural antioxidant flavonolignans silymarin components silybin A/B represent a particular lipase substrate, showing a broad range of pharmacological properties [47]. The therapeutic properties of silybin are directly related to its antioxidant (radical scavenging), detoxifying, and regenerative activities, providing broad hypocholesterolemic, cardioprotective, and neuroprotective effects [48,49,50,51]. 

Considering that 30–60% of the silymarin remains in the milk thistle cake during industrial cold-pressed oil production [52,53], valorization of the waste biomass resulting from this process is highly beneficial for the agro-food domain. For that, the identification of a potent cold-active lipase biocatalyst dedicated to silybin A/B acylation with fatty acid esters is of particular interest, considering its high stability and activity at low and moderate temperatures.

In this context, the current study focused on obtaining and characterizing a cold-active recombinant lipase (PSL2) from the ice cave psychrophilic *Psychrobacter SC65A.3* bacterial strain, testing its ability as a biocatalyst for silybin acylation. 

## 2. Results and Discussion

### 2.1. Primary Structure Analysis of PSL2 from Psychrobacter SC65A.3

Screening of the recently determined genome sequence of the cave ice bacterial strain *Psychrobacter* SC65A.3 [46] revealed a 1449 bp coding region (accession number OQ547793.1) homologous to alpha/beta hydrolases, corresponding to a 483-amino acid hypothetical lipase (PSL2) with a calculated molecular weight of 53626.97 Da and a theoretical pI of 5.33 (Supplementary Figure S1). 

A BLAST analysis of the PSL2 amino acid sequence [WP_263241157.1] indicated that this cold-active enzyme is homologous (99% identity) to the alpha/beta hydrolase [WP 020444543] of the Antarctic *Psychrobacter* sp. G strain [54]. Meanwhile, a very low identity (29.9–35.3%) and similarity (47–49%) were observed with microbial lipases from psychrophilic, mesophilic, and (hyper)thermophilic species, showing no correlation with the growth temperature of the host (Table 1).

The primary structure of PSL2 consisted of 54% hydrophobic, 46% polar, 12% acidic, and 8% basic amino acids (Supplementary Table S1). Notably, the reduced content of bulky aromatic residues (7%) compared to aliphatic residues (44%) may contribute to the flexibility of the enzyme at low temperatures [55,56]. 

The lower content of small amino acids (Ala and Gly) in PSL2 (14.3%) relative to that in homologous enzymes from the mesophilic *B. subtilis* (18.2%) and *P. aeruginosa* (21.3%) and the thermophilic *B. thermocatenalatus* (18.8%) (Table 1) is expected to improve the protein stability at low temperatures by decreasing the number of hydrogen bonds [57]. Meanwhile, the comparable content of these residues (12.3%) in the *S. islandicus* lipase may be attributed to the positioning within the secondary structure and the accessibility of these amino acids in the hyperthermophilic homolog [57]. Also, the significantly higher contents of Pro, His, and Met residues in PSL2 and other cold-active lipases, compared to their mesophilic and thermophilic counterparts (Table 1), provide further structural evidence supporting the enzyme’s adaptation to cold environments [42,58,59,60]. Moreover, the calculated ratio of Arg/Arg + Lys residues (Table 1) used as an evaluation marker for the formation of protein salt bridges [57,61] showed a relatively lower value in the case of the cold-adapted PSL2 (0.39) in comparison with that of the mesophilic homologs (0.47–0.53) and the thermophilic *B. thermocatenulatus* (0.67), corresponding to a reduced number of ion interactions that could assess for a higher flexibility for catalysis at low temperatures. However, a similarly low score was observed for the hyperthermophilic *S. islandicus* lipase, suggesting their different implications for structural stability, given the very low sequence identity of these enzymes.

**Figure 1:**
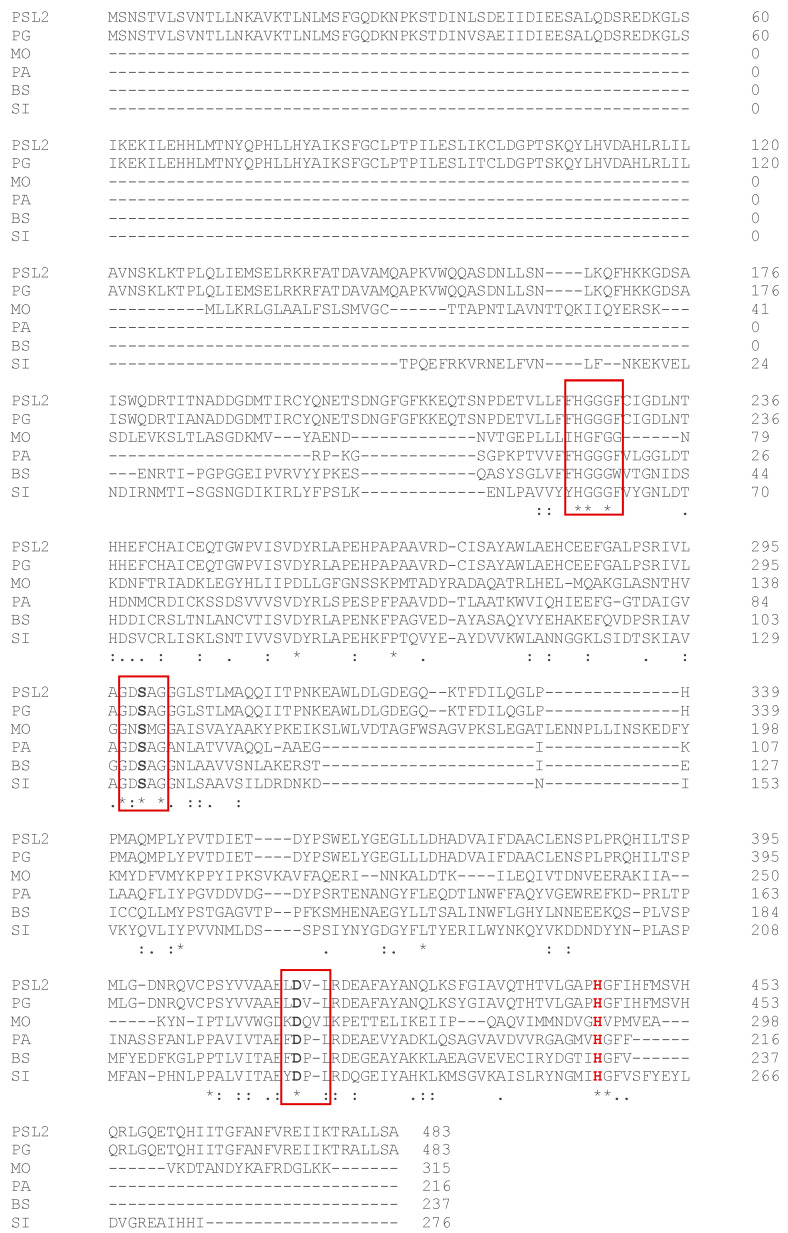
Multiple alignment of the primary PSL2 sequence with homologous enzymes from the psychrophilic *Psychrobacter* sp. G [WP 020444543] (PG) and *Moritella* sp. PE36 [WP 198138589] (MO), the mesophilic *Bacillus subtilis* [WP 161476533] (BS) and *Pseudomonas aeruginosa* [EIU5571796] (PA), and the hyperthermophilic *Sulfolobus islandicus* [WP 015580697] (SI). Identical (stars) and conserved (dots) residues from all enzymes, active site residues (bold), catalytic His444 (red), and conserved motifs (boxes) are shown. Amino acid numbers for each enzyme primary structure are indicated.

A multiple alignment of the cold-active PSL2 with homologs from the psychrophilic *Psychrobacter* sp. G [WP 020444543] and *Moritella* sp. PE36 [WP 198138589], mesophilic *Bacillus subtilis* [WP 161476533] and *Pseudomonas aeruginosa* [EIU5571796], and the hyperthermophilic *Sulfolobus islandicus* [WP 015580697] revealed the presence of conserved motifs and catalytic residues specific for family IV lipolytic enzymes [7] in the ice cave lipase (Figure 1). These conserved active site elements comprised the hydrophobic HGGGF motif (residues 225–229) [30], the nucleophilic Ser299-containing GDSAG motif (residues 297–301), an acidic residue (D414) in a consensus sequence LDXL, and a catalytic histidine (H444), in support of an active PSL2 lipase (Figure 1). This classification was also confirmed by the presence of the catalytic Ser-His-Asp triad and the high sequence identity (99%) with the *Psychrobacter* sp. G enzyme that belongs to the hormone-sensitive lipase (HSL) group [62]. 

### 2.2. Cloning, Expression, and Purification of PSL2

The PSL2 coding gene (*lip*2, accession number OQ547793.1) identified in the *Psychrobacter* SC65A3.1 genome sequence corresponding to the putative lipase PSL2 (Supplementary Figure S1) was amplified and cloned into the pHAT2 expression vector using the *Nco*I/*Bam*HI cloning sites (ATG: Biosynthetics GmbH, Merzhausen, Germany). Heterologous gene expression of this construct (pPSL2) in *Escherichia coli* BL21 (DE3) tested at different temperatures (15 °C, 25 °C, and 37 °C) was optimal after induction at 25 °C for 16 h in the presence of 0.5 mM IPTG (Supplementary Figure S2). The His-tag motif appended to the amino end of the recombinant enzyme allowed the purification of the enzyme from the soluble fraction by Ni^2+^-NTA affinity chromatography, with elution by 100 mM imidazole in 100 mM Tris HCl, pH 8 (Supplementary Figure S2). The production yield of the purified recombinant PSL2 was 12.08 ± 1.72 µg L^−1^ of culture. The specific activity of the new cold-active lipase measured at 25 °C in the presence of 1 mM *p*-NPB and 50 mM Tris HCl pH 7.2 buffer was 20.1 ± 3.2 U mg^−1^.

### 2.3. Functional Characterization of the Recombinant PSL2

#### 2.3.1. Effects of pH and Solvents

**Figure 2:**
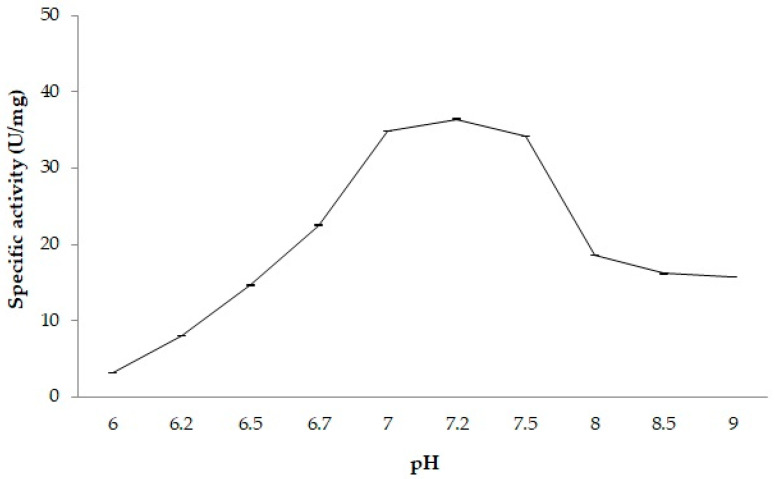
pH effect on the PSL2 activity. The lipase activity was measured at 25 °C in the presence of 2.5 mM *p*-NPB and 50 mM PBS (pH 6.0–7.0) or 50 mM Tris HCl buffer (pH 7.0–9.0) using 51 µg of PSL2, as indicated in the Methods. Average and standard deviation values of specific activities were calculated based on triplicate measurements.

The activity of PSL2 was assessed over a pH range of 6.0 to 9.0 (Figure 2), showing higher values (23–36 U mg⁻¹) in the pH range of 7.0–7.5, with a peak at pH 7.2. A partial inactivation of 53.85 ± 4.07% was observed when the reaction occurred within the pH range of 8.0–9.0, while enzymatic activity increased in the pH 6.0–7.0 interval (Figure 2). Consequently, pH 7.2 was determined as the optimal pH for the lipolytic activity of the recombinant PSL2.

In comparison to other homologous enzymes from *Psychrobacter* species found in cold environments, PSL2 exhibited a neutral optimal pH, while both EstPc esterase [63] and Lip 1PC lipase [64] from the psychrophilic *P. cryohalolentis* K5T isolated from Siberian permafrost showed the highest activity in a slightly alkaline environment (pH 8.5). Similarly, lipases of other *Psychrobacter* strains originating from Arctic (pH 8) [65] and Antarctic seawater (pH 8) [66], as well as deep-sea sediments (pH 9) [3], also favored alkaline conditions. Moreover, the lipase isolated from the psychrophilic *Acinetobacter* sp. XMZ-26 strain retrieved from glacier soil in China was highly active (>80%) in the pH range of 8–11, with an optimum at pH 10 [67]. A relatively neutral pH was also observed in the case of LSK25 lipase from a psychrophilic *Pseudomonas* strain collected from Antarctic soil, which was active in the pH 6–8 interval, with an optimum at pH 6.0 [68]. Additionally, the thermophilic *Bacillus halodurans* from Egyptian soil exhibited an optimum pH in the range of 7–8 [69]. This functional characteristic aligns with the structural variability of these extremozymes and is independent of the temperature of their habitats. 

**Figure 3:**
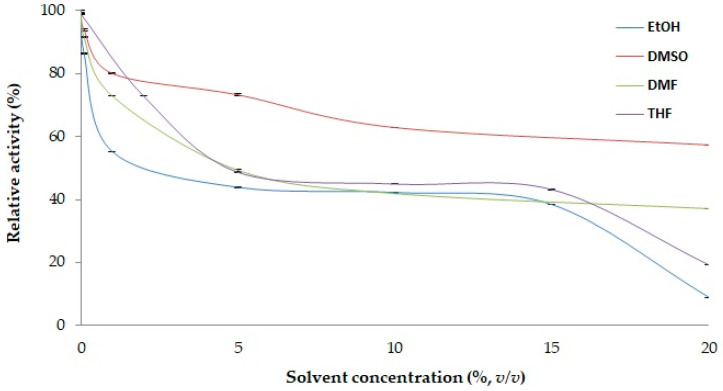
Solvent tolerance stability of PSL2. The lipase activity was measured at 25 °C using 40.8 µg of PSL2 and 3.75 mM *p*-NPB as the substrate in the presence of ethanol (EtOH), dimethyl sulfoxide (DMSO), dimethylformamide (DMF), and tetrahydrofuran (THF) at final concentrations ranging from 0% to 20% (*v/v*). The relative activity in the absence of solvent was calculated as 100%. Average and standard deviation values of specific activities were calculated based on triplicate measurements.

In the context of the limited organic solvent tolerance of lipases [70], four different organic solvents—EtOH, DMSO, DMF and THF—were tested at concentrations ranging from 0 to 20% (*v/v*) to evaluate their effect on PSL2 activity. The results are shown in Figure 3. PSL2 activity was partially inhibited (by ~50%) in the presence of 5–15% solvents. The negative effect of solvents on PSL2 activity appeared to be a specific characteristic of cold-active lipases [6]. Notably, DMSO had a moderate inhibitory effect, preserving 73% and 57% of PSL2 activity at concentrations of 5% and 20% solvent, respectively. This trend was similar to that of other cold-active lipases, including those from the permafrost-originating *Psychrobacter* sp. ZC12 [65], EstPc [63], and Lip1Pc [64], which demonstrated high stability in up at least 10% of this solvent and partial inactivation of up to 67% in 5–10% ethanol. However, the psychrotolerant *Psychrobacter* Lip1PC lipase appeared to be highly unstable in DMF, with 2.5% and <0.5% residual activity in the presence of 5% and 10% solvent, respectively [64]. In contrast, PSL2 displayed a higher stability to ethanol than the cold-active lipase from the Antarctic *Pseudomonas* sp. LSK25, which was completely inactivated by this solvent [68].

As the acylation of silybin by PSL2 lipase required substrate solubilization in THF, this solvent was selected as the reaction medium due to its effectiveness in dissolving the substrate, reagents, and products. The solubility of silybin in THF was shown to exceed 140–150 mg/mL [71], and both methyl esters and fatty acids such as methyl palmitate with the longest chains are fully soluble in THF [72], with a solubility of 1 g/mL [73]. Under these conditions, PSL2 retained approximately 40% of its activity in the presence of 5–15% THF solvent (Figure 3).

#### 2.3.2. Substrate Specificity

**Figure 4:**
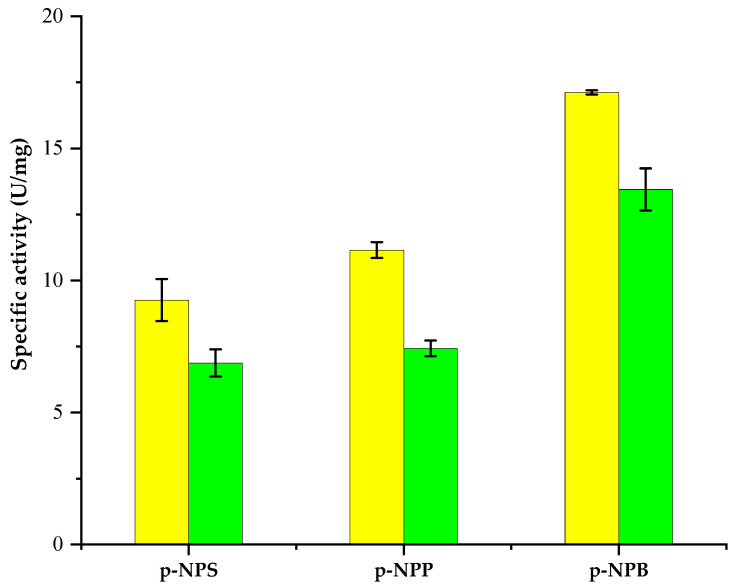
Substrate specificity of PSL2. The specific activity was measured at 25 °C (yellow) and 40 °C (green), as indicated in the Methods, using *p*-nitrophenyl stearate (*p*-NPS), *p*-nitrophenyl palmitate (*p*-NPP), and *p*-nitrophenyl butyrate (*p*-NPB) as the substrates and 16.8 µg of PSL2. Average and standard deviation values of specific activities were calculated based on triplicate measurements.

The substrate specificity of PSL2 was evaluated using *p*-NPS, *p*-NPP, and *p*-NPB (Figure 4). The results showed that this cold-active enzyme could recognize various *p*–nitrophenyl esters as substrates at both low (25 °C) and high (40 °C) temperatures, with a preference for the short-chain compound (C4) (Figure 4). For long carbon chain esters (C14 and C16), the PSL2 activity measured at 25 °C was 1.2-fold and 1.8-fold lower, respectively, as compared with that for the short carbon chain (C4) substrate, with both activities reduced by 1.4-fold when measured at 40 °C (Figure 4). Moreover, the lower temperature resulted in a significant increase in enzyme activity (by 26.8 ± 4.9%) independent of the substrate (Figure 4), further confirming the cold-active nature of the PSL2 lipase.

Unlike the homologous enzyme Lip1PC from the psychrotrophic *P. criohalolentis* K5T for which the lipolytic activity towards *p*-NPP represented only 2% of the maximum activity measured for *p*-NPB [64], PSL2 showed a relatively high activity when using long-chain fatty acids, representing 65% and 54% for *p*-NPP- and *p*-NPS-catalyzed reactions, respectively (Figure 4). 

Other extremophilic lipases tested previously demonstrated catalytic recognition for medium-chain esters, a characteristic feature of GX class lipases [6].

#### 2.3.3. Thermal Stability

**Figure 5:**
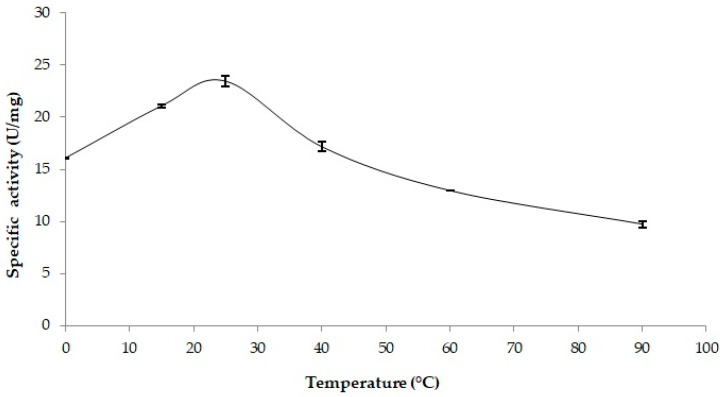
Thermal stability of PSL2. The enzyme (4 µg) was incubated at various temperatures in the 4 °C–90 °C interval for 30 min and the activity was measured at 25 °C using *p*-NPP as the substrate (see the Methods). The relative activity in the absence of thermal treatment was calculated as 100%. Average and standard deviation values of specific activities were calculated based on triplicate measurements.

The thermal stability of PSL2 was assessed by incubating the enzyme for 30 min at various temperatures ranging from 4 °C to 90 °C, followed by measuring the residual activity at 25 °C under standard conditions (Section 3.4). Under these conditions, PSL2 preserved full activity up to 40 °C, with an apparent activation of 45.7% at 25 °C relative to that of the untreated enzyme (Figure 5). Notably, this cold-active enzyme showed a residual activity of 80.6% after incubation at 60 °C, and retained 60.7% of lipolytic activity at 90 °C, demonstrating a particularly high thermal stability, similar to that of the hyperthermophilic lipase homolog [30] and of other extremozymes adapted to high temperatures [71]. Meanwhile, some cold-active lipases, such as CalA from *Candida antarctica*, have been reported to exhibit a remarkable thermal stability, with an optimal temperature exceeding 90 °C [74].

Considering that most of the reported cold-active lipases are heat-labile [42,55], the perform high catalytic activity of PSL2 at temperatures up to 90 °C represent an important advantage for biotechnological and industrial applications [72]. Unlike other *Psychrobacter* lipases showing rapid inactivation above 35 °C [63,73] and the versatile TLL biocatalyst from *Thermomyces lanuginosus* commercially used in the oleochemical industry that preserved only 40% residual activity at 55 °C [75], the recombinant ice cave enzyme appeared to be a highly thermostable putative biocatalyst.

#### 2.3.4. Kinetic Parameters

To evaluate the catalytic efficiency of PSL2 for the hydrolysis of *p*-NPB, *p*-NPP, and *p*-NPS, substrate saturation curves were carried out at 25 °C and used to calculate the steady-state kinetic parameters (Table 2). The K_m_ values for these substrates ranged from 2.38 mM to 4.30 mM, indicating a 1.5-fold and 1.8-fold higher apparent affinity for the short chain (butyrate) phenyl ester as compared to the long chain (stearate and palmitate) derivatives, respectively (Table 2). Comparable reaction rate (k_cat_) values were obtained for the butyrate (3.089 min^−1^) and stearate (3.564 min^−1^) derivatives, while the rate for palmitate acylation was about 2-fold lower (1.482 min^−1^). The corresponding catalytic efficiency indicated a preference for the esterase reaction of PSL2 when using *p*-NPB (1.497 mM^−1^ min^−1^). In this case, the catalytic efficiency was 1.8-fold and 4.3-fold higher than the lipolytic activity for the *p*-NPS and *p*-NPP derivatives, respectively (Table 2).

Cold-active lipases are known for their high catalytic efficiency at low and moderate temperatures [25,41,42]. Among these, the ZC12 lipase originating from the Arctic *Psychrobacter* sp. ZY124 strain exhibited a 3-fold lower apparent affinity (K_m_ 13.1 mM) for *p*-NPP and 4.4-fold higher catalytic efficiency than PSL2 [65]. 

In comparison with other lipases used as biocatalysts for silybin acylation, PSL2 demonstrated a more efficient catalysis at 25 °C, with a 65-fold higher apparent affinity (K_m_ = 280.8 mM), and only 5-fold lower catalytic efficiency (0.026 mM^−1^ s^−1^) for *p*-NPP hydrolysis as compared to the soluble *Pseudomonas cepacia* lipase at 37 °C [76]. Moreover, both free and immobilized lipase-B from *Candida antarctica* showed comparable K_m_ values of 3.24 mM and 4.36 mM, respectively, for *p*-NPP hydrolysis [77], further supporting the PSL2 potential as an efficient catalyst.

### 2.4. Silybin Acylation Using PSL2

**Figure 6:**
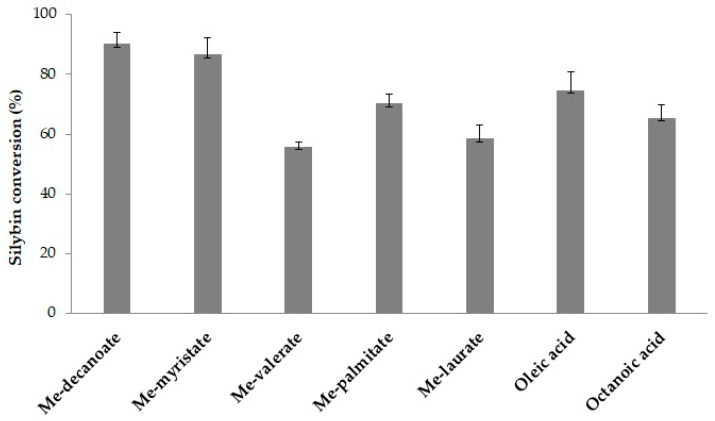
Influence of the acylation reagents on the silybin derivatization. Experimental conditions: 2 mM silybin, acylation reagent, and 0.31% PSL2 lipase (*v*/*v*) in THF; 1000 rpm, 24 h, 25 °C. Average and standard deviation values of specific activities were calculated based on triplicate measurements.

The catalytic activity of recombinant PSL2 lipase was evaluated for silybin acylation with fatty acids (FA) and fatty acids methyl esters (FAMEs). Different acylation reagents were tested, including Me-decanoate, Me-myristate, Me-valerate, Me-palmitate, Me-laurate, oleic acid, and octanoic acid, in order to produce silybin derivatives. The experimental results are shown in Figure 6. As a general remark, all the tested reagents achieved more than 50% conversion of silybin. Specifically, maximum substrate conversions of 90% and 87% were reached for Me-decanoate and Me-myristate, respectively, while methyl valerate resulted in the lowest conversion of silybin (56%). This indicated that the PLS2 lipase preferentially recognized acylation reagents with larger carbon chains. Based on these findings, Me-decanoate was selected as the acylation reagent for subsequent experiments.

**Figure 7:**
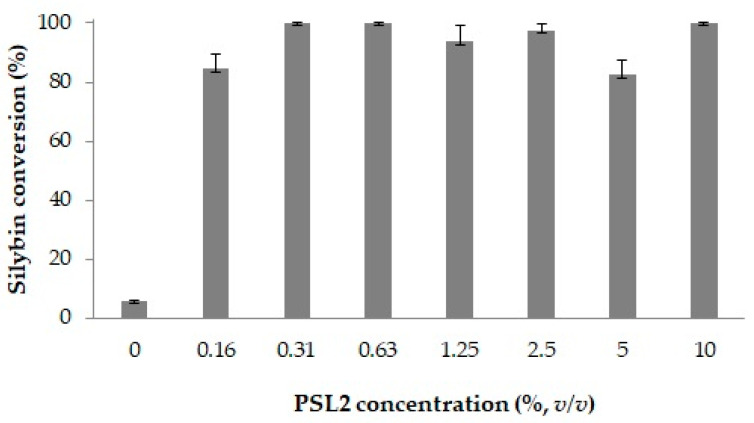
The effect of the PSL2 concentration on the silybin acylation. Experimental conditions: 2 mM silybin, 45 mM Me-decanoate, and PSL2 lipase in THF; 1000 rpm, 24 h, 25 °C. Average and standard deviation values of specific activities were calculated based on triplicate measurements.

The effect of the catalyst concentration on silybin acylation assisted by PLS2 was evaluated across a range of 0–10% (*v*/*v*) cold-active lipase. The experimental results are shown in Figure 7. An increasing trend in silybin conversion was observed when the enzyme concentration increased from 0 to 0.31%. However, further increases in the enzyme concentration did not significantly affect the conversion rate. A consistent conversion rate was noticed in the range 0.31–10% (*v*/*v*). Since a concentration of 0.31% PSL2 lipase achieved the maximum conversion of 90% silybin, this concentration of biocatalyst was selected for subsequent experiments.

**Figure 8:**
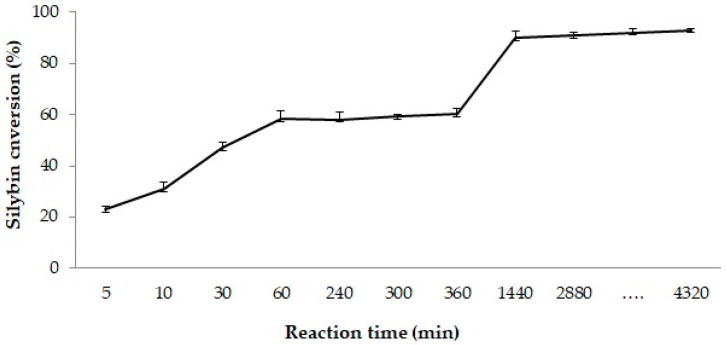
Variation of the reaction time for silybin acylation. Experimental conditions: 2 mM silybin in THF, 45 mM Me-decanoate, and 0.31% PSL2 lipase (*v/v*), 1000 rpm, 25 °C.

To evaluate the overall performance of the biocatalytic silybin acylation, identical reaction mixtures were prepared and incubated under the same experimental conditions, with samples analyzed at different time points ranging from 0 to 4320 min (Figure 8). During the first hour of the reaction, silybin conversion increased continuously from 53% to 58%. Subsequently, the substrate conversion remained constant for the next 5 h (58–59%). The maximum transformation of silybin (90%) was achieved after 24 h and this conversion level was maintained for 72 h of incubation time. The two increasing trends in silybin conversion showed distinct slopes (Figure 8). This behavior may be attributed to a different recognition for silybin A and B diastereoisomers from the substrate mixture used in the acylation reaction. In this respect, the use of *Candida antarctica* lipase was previously reported to separate the diastereoisomers of silybin based on a similar recognition pattern [78].

Our previous data from the investigation of silybin acylation using extracellular fractions of *Psychrobacter* SC65A.3 based on a similar protocol [39] indicated a maximum conversion of 19% for the methyl decanoate agent at 25 °C for a 24 h (1440 min) reaction time. Thus, the use of the purified recombinant PSL2 enzyme resulted in an increase in silybin conversion by more than 4-fold under similar experimental conditions. This is clear evidence that PSL2 can be considered a valuable biocatalyst for silybin derivatization.

## 3. Materials and Methods

### 3.1. Reagents 

Silybin (mixture of diastereoisomers silybin A and B) and Tris HCl buffer (2-amino-2-(hydroxymethyl)-1,3 propanediol hydrochloride) were purchased from Sigma–Aldrich (Taufkirchen, Germany). Ethanol (EtOH), dimethyl sulfoxide (DMSO), dimethylformamide (DMF), tetrahydrofuran (THF), bovine serum albumin, *p*-nitrophenyl stearate (*p*-NPS), *p*-nitrophenyl palmitate (*p*-NPP), *p*-nitrophenyl butyrate (*p*-NPB), oleic acid, octanoic acid, *p*-nitrophenol, and sodium bicarbonate were from Sigma–Aldrich–Merck, Darmstadt, Germany. Isopropyl-β-D-thiogalactoside (IPTG) was obtained from Carl Roth, Karlsruhe, Germany.

### 3.2. Cloning and Heterologous Expression of the Lip2 Gene from Psychrobacter SC65A.3 Genome

The DNA sequence of the *lip2* (1449 bp) gene (Supplementary Figure S1) identified in the *Psychrobacter* SC65A.3 genome (accession number CP106752.1) was synthesized and cloned into the pHAT2 His-tag expression vector (EMBL, Heidelberg, Germany) using the *Nco*I/*Bam*HI restriction sites (ATG Biosynthetics GmbH, Merzhausen, Germany). The expression of the resulting recombinant plasmid was carried out for 16 h at 15 °C, 25 °C and 37 °C in *Escherichia coli* BL21(DE3) (Thermo Fisher Scientific, Waltham, MA, USA) after induction with 0.5 mM IPTG in Luria–Bertani LB liquid medium [79]. The cells were collected by centrifugation at 9000× *g* for 10 min under refrigeration (4 °C), and stored at −80 °C. 

### 3.3. Purification of Recombinant PSL2 

The psychrophilic recombinant enzyme PSL2 was purified using an adapted affinity chromatography method based on Ni-NTA (Qiagen, Hilden, Germany) [80]. The induced *E. coli* cells (200 mL culture) were resuspended in 6 mL of buffer TN (100 mM Tris HCl, pH 8, 200 mM NaCl) and disrupted by a 5 min treatment alternating cycles of 5 s pulses and 60 s pauses using a Sonopuls ultrasonic homogenizer (Bandelin, Berlin, Germany). The soluble fraction that resulted after centrifugation at 16,000× *g* for 30 min at 4 °C was applied to a 1 mL Ni-NTA agarose (Qiagen, Hilden, Germany) column equilibrated with buffer TN. The column was washed with buffer TN (10 mL) and buffer A containing 30 mM imidazole (5 mL), and the recombinant protein was eluted in the presence of 100 mM imidazole. After analysis by SDS-PAGE (Bio-Rad, Berkeley, CA, USA) the protein fractions containing PSL2 were desalted using 7K MWCO Zeba Spin Desalting columns (Thermo Fisher Scientific, Waltham, MA, USA). The purified enzyme was stored at −20 °C in 100 mM Tris HCl buffer, pH 8, containing 20% glycerol. 

### 3.4. Lipase Assays

The activity of SPL2 lipase was measured based on the spectrophotometric method [81] using *p*-nitrophenylbutyrate (*p*-NPB) in ethanol (EtOH) as solvent. The reaction was performed at 25 °C or alternatively at 40 °C. The enzyme (4 μg) was incubated for 30 min with 0.125 mM substrate and 50 mM Tris HCl, pH 7.2, and the reaction was stopped after an incubation for 10 min with a 20 mM Na_2_CO_3_ blocking solution. The absorbance of the reaction product was measured at 347 nm using a Specord 250 spectrometer (Analytic Jena, Wien, Austria), and the lipase activity was calculated based on a calibration curve obtained for a range of 0.1 mM–0.5 mM *p*-nitrophenol in ethanol. The enzymatic activity was expressed in U mg⁻¹ protein, with one unit (U) being defined as μM min⁻¹. 

The optimal pH was determined at 25 °C in the presence of 50 mM potassium phosphate buffer (PBS; pH 6.0, 6.2, 6.5, 6.7, or 7.0) and 50 Tris HCl buffer (pH 7.0, 7.2, 7.5, 8.0, 8.5, or 9.0), using 2.5 mM *p*-NPB as substrate.

The solvent tolerance of SPL2 was evaluated by measuring the PSL2 lipolytic activity at 25 °C using *p*-NPB as the substrate in the presence of 1–20% (*v*/*v*) EtOH, dimethyl sulfoxide (DMSO), dimethylformamide (DMF), or tetrahydrofuran (TMF). 

The thermal stability was determined by incubating the enzyme at various temperatures in the 4 °C–90 °C interval (4 °C, 15 °C, 25 °C, 40 °C, 60 °C, and 90 °C) for 30 min, and measuring the PSL2 residual activity at 25°C under standard conditions using *p*-NPB as substrate. 

Substrate specificity was determined at 25 °C and at 40 °C using 1 mM *p*-NPB, *p*-NPP, and *p*-NPS as substrates. 

The steady-state kinetic parameters K_M_ and V_max_ were calculated from substrate saturation curves carried out at 25 °C in the presence of variable concentrations of substrates by fitting the experimental data to the Michaelis–Menten equation. 

### 3.5. Sequence Analyses 

The analysis of the amino acid composition of the hypothetical lipase PSL2 and calculations of the theoretical molecular weight (MW) and isoelectric point (pI) were performed using the ExPASy ProtParam platform (https://web.expasy.org/cgi-bin/protparam/protparam) (accessed on 25 February 2021) [82]. Sequence similarity and identity percentages of the cave enzyme and homologous bacterial lipases were determined using the Emboss Needle pair alignment tool (http://www.ebi.ac.uk/Tools/psa/emboss_needle) (accessed on 7 August 2022) [83]. Multiple alignment of lipase primary sequences was performed using the CLUSTAL OMEGA EMBL-EBI (1.2.4) platform (https://www.ebi.ac.uk/Tools/msa/clustalo/) (accessed on 16 September 2022) [83].

### 3.6. Biocatalytic Acylation of Silybin 

For silybin acylation, the reaction mixture was prepared and contained 2 mM silybin and 45 mM Me-decanoate/40 mM Me-laurate/35 mM Me-myristate/30 mM Me-palmitate, and 10% PSL2 lipase (*v*/*v*) in THF. The mixture was incubated at 25 °C and stirred at 1000 rpm for 24 h. After centrifugation for 15 min at 1500 rpm, the supernatant was filtered using 0.22 μm Millipore filters and dried at 70 °C to evaporate the solvent. The dried samples were resuspended in a solvent mixture (41:59 acetonitrile:acetone) and analyzed by HPLC. For that, a 1260 Infinity HPLC modular system equipped with a Poroshell 120 EC-C18 column and a diode array-type detector (DAD) was used (Agilent Technologies, Waldbronn, Germany). The analysis conditions were a mobile phase (41:59 acetonitrile:acetone) with flow rate of 1 mL/min injection volume of 25 μL of sample, detector wavelength of 210 nm, and analysis time of 30 min. Identification of the sample components (silybin and acylation reagents) was performed using specific standards.

## 4. Conclusions

A cold-active recombinant lipase (PSL2) from a *Psychrobacter* SC65A.3 strain isolated from Scarisoara Ice Cave (Romania) was successfully produced, yielding 12.08 ± 1.72 µg L^−1^ of culture and exhibiting a lipolytic activity of 20.1 ± 3.2 U mg^−1^. Structural and functional characterization confirmed the cold-active nature of PSL2. Notably, this enzyme originating from a bacterium recovered from 900-year-old ice exhibited a particularly high thermal stability, maintaining full activity up to 40 °C and retaining more than 60% residual activity after an incubation at 90 °C. Kinetic tests further demonstrated the ability of PSL2 to catalyze esterase reactions at 25 °C, supporting its cold-active properties.

Recombinant PSL2 was successfully tested as a cold-active biocatalyst for silybin acylation with methyl fatty acid esters (e.g., Me-decanoate, Me-myristate, Me-valerate, Me-palmitate, and Me-laurate) and fatty acids (e.g., oleic acid and octanoic acid). Conversion rates exceeded 50% for all acylation reagents, with a maximum of 90% for Me-decanoate, highlighting the potential of PSL2 as an effective biocatalyst for silybin acylation.

The biocatalytic efficiency of the cold-active PSL2 lipase for silybin acylation offers a significant advantage for recovering silybin from milk thistle cake, a byproduct (waste biomass) of cold-pressed milk thistle oil production. Moreover, silybin derivatization with fatty acid residues leads to more hydrophobic derivatives. Thus, the cold-active biocatalytic behavior of PSL2 aligns with green chemistry concepts by enabling energy-efficient acylation processes.

These corroborated features recommend the cold-active PSL2 lipase as a valuable alternative for industry, enhancing the technological process of cold-pressed milk thistle oil production. Additionally, cost-effective biocatalyst design is currently under development in our lab, with a focus on immobilizing PSL2 lipase to obtain a recyclable biocatalyst. The system’s performance of PSL2 activity and regio/enantio-selectivity under optimum experimental conditions will also be assessed to evaluate the potential of this cold-active lipase in biotechnologies. 

## Figures and Tables

**Table 1:** Homology and amino acid composition.

Extremophiles	Lipase Homologs [Accession Numbers]	Identity (%)	Similarity (%)		Amino Acid Content (%) Ratio			
				Gly + Ala	Pro	His	Met	Arg/ (Arg + Lys)
Psychrophiles	*Psychrobacter* SC65A.3 PSL2 [WP_263241157.1]	100	100	14.3	5	4.3	2.3	0.39
	*Psychrobacter* sp. G [WP 020444543]	98.9	99	12.7	5	4.3	2.3	0.41
	*Moritella* sp. PE36 [WP 198138589]	27.5	44	13.4	4.4	4.4	1.8	0.48
Mesophiles	*Bacillus subtilis * [WP 161476533]	32.5	49	18.2	6.3	2.5	1.3	0.47
	*Pseudomonas aeruginosa* [EIU5571796]	35.3	48	21.3	6	1.9	0.9	0.53
Hyper/ thermophiles	*Bacillus thermocatenalatus * [WP 253270889]	NS	NS	18.8	4.8	3.1	2.2	0.67
	*Sulfolobus islandicus* [015580697]	29.9	49	12.3	4.7	2.9	1.8	0.39

NS: non-significant similarity.

**Table 2:** Steady state kinetic parameters of PSL2 measured at 25 °C.

Substrate	K_m_ (mM)	V_max_ (U mg^−1^)	k_cat_ (min^−1^)	k_cat_/K_m_ (mM^−1^ min^−1^)
*p*-NPB	2.38	37.88	3.564	1.497
*p*-NPP	4.30	15.75	1.482	0.345
*p*-NPS	3.70	32.82	3.089	0.835

## Data Availability

All data supporting the conclusions of this article are included in the manuscript. The annotated genome sequence of *Psychrobacter* SC65A.3 was deposited in GenBank under the accession number CP106752.

## Supplementary Materials

The following supporting information can be downloaded at https://www.mdpi.com/article/10.3390/molecules29215125/s1: Figure S1. *Psychrobacter* SC65A3.1 *lip2* gene nucleotide sequence and corresponding amino acid sequence of PSL2 lipase; Table S1. Amino acid composition of PSL2; Figure S2. Purification of recombinant PSL2 by Ni-NTA affinity chromatography.
