# Supplementary material for: Cold-Active Lipase from the Ice Cave Psychrobacter SC65A.3 Strain, a Promising Biocatalyst for Silybin Acylation
Source: Molecules. 2024 Oct 30;29(21):5125. doi: 10.3390/molecules29215125 (PMC11547725; doi:10.3390/molecules29215125)
Supplement: Supplementary file 1 [file molecules-29-05125-s001.zip › molecules-3255217-supplementary.pdf]

molecules

Supplementary Materials

Cold-active lipase from the ice cave *Psychrobacter* SC65A.3 strain, promising biocatalyst for silybin acylation

Victoria I. Paun [1#], Sabina Gabriela Ion [2#], Giulia R. Gheorghita [1], Iunia Podolean [2], Madalina Tudorache [2*], Cristina Purcarea [1*]

[1] Department of Microbiology, Institute of Biology Bucharest of the Romanian Academy, 296 Splaiul Independentei, 060031 Bucharest, Romania; ioana.paun@ibiol.ro (VIP); gheorghita.giulia@yahoo.com (GRG)

[2] Department of Anorganic Chemistry, Organic, Biochemistry and Catalysis, Faculty of Chemistry, University of Bucharest, 4-12 Regina Elisabeta Blvd., 030016 Bucharest, Romania; ion.sabina.gabriela@chimie.unibuc.ro (SA); iunia.podolean@chimie.unibuc.ro (IP)

* Correspondence: cristina.purcarea@ibiol.ro ; +40746151081 (CP), madalina.sandulescu@g.unibuc.ro (MT)

# authors with equal contribution

Psychrobacter SC65A3.1 *lip*2 gene [OQ547793.1]

ATGTCTAACTCAACAGTACTATCAGTCAACACCTTACTCAATAAAGCGGTGAA-
GACCCTAAATCTTATGTCATTTGGACAAGATAAGAACCCCAAAAGCACTGATATCAATTTATCAGATGAAATAATAGATATAGAA
GAGAGTGCTTTGCAAGATAGTCGCGAGGATAAAGGCTTATCTATTAAAGAAAAGATACTT-
GAGCACCATCTGATGACCAACTATCAACCGCATCTACTGCACTATGCCATAAAAAGCTTTGGCTGCTTACCTACGCCGATTCTTGA
GAGTTTGATAAAGTGTTTAGATGGACCTACTTCAAAGCAATATTTGCATGTT-
GATGCCCATCTGCGCTTAATACTTGCGGTCAATAGCAAGCTAAAAACGCCTTTGCAGCTGATAGAGATGTCTGAGCTGCGTAAAC
GCTTTGCAACAGATGCGGTCGCTATGCAAGCACCAAAAGTATGGCAGCAA-
GCTTCTGACAACTTGCTTAGCAATTTGAAACAGTTTCATAAAAAAGGCGATAGCGCTATCAGTTGGCAAGATAGAACGATTACCA
ATGCCGACGACGGTGATATGACCATTCGCTGCTATCAAAATGAGACCTCTGACAATGGTTTT-
GGCTTTAAAAAAGAGCAAACCAGTAATCCTGATGAGACCGTGCTATTGTTTTTTCATGGTGGTGGATTTTGTATTGGTGACCTTA
ATACCCATCATGAATTTTGTCATGCCATTTGTGAGCAAACGGGCTGGCCGGTCATTAGTGTT-
GATTATCGCTTAGCACCTGAACACCCTGCCCCTGCTGCCGTAAGAGACTGTATCAGTGCCTATGCTTGGTTAGCTGAACATTGTGA
AGAATTTGGTGCTTTACCATCACGCATTGTATTAGCAGGTGATAGCGCAGGTGGTGGAT-
TATCTACTTTGATGGCTCAGCAAATCATCACGCCAAATAAAGAGGCTTGGTTGGATCTAGGTGATGAAGGTCAAAAGACCTTTGA
TATATTACAAGGTTTACCACATCCTATGGCACAGATGCCCTTATATCCTGTGACCGA-
TATTGAGACCGATTATCCAAGCTGGGAGTTATATGGTGAAGGTCTATTACTCGACCATGCTGATGTTGCCATTTTTGATGCCGCTT
GTTTAGAAAATAGTCCGTTACCGCGCCAGCATATCCTGACCTCACCGATGCTTGGG-
GACAATCGACAGGTTTGTCCAAGTTATGTCGTTGCAGCAGAATTAGATGTCTTACGTGATGAAGCGTTTGCTTATGCCAATCAGC
TAAAGAGTTTTGGTATAGCCGTACAAACCCATACGGTACTTGGCGCACCGCATGGAT-
TTATTCACTTTATGAGCGTTCATCAAAGATTAGGGCAAGAAACTCAGCATATCATCACAGGGTTTGCTAATTTTGTGCGTGAAATC
ATAAAAACAAGGGCGCTATTGAGCGCTTAA

PSL2 Lipase [WP_263241157.1]

MSNSTVLSVNTLLNKAVKTLNLMSFGQDKNPKSTDINLSDEIIDIEESALQDSREDKGL-
SIKEKILEHHLMTNYQPHLLHYAIKSFGCLPTPILESLIKCLDGPTSKQYLHVDAHLRLILAVNSKLKTPLQLIEMSELRKRFATDAVAMQA
PKVWQQASDNLLSNLKQFHKKGDSAISWQDRTITNADDGDMTIR-
CYQNETSDNGFGFKKEQTSNPDETVLLFFHGGGFCIGDLNTHHEFCHAICEQTGWPVISVDYRLAPEHPAPAAVRDCISAYAWLAEHC
EEFGALPSRIVLAGDSAGGGLSTLMAQQIITPNKEAWLDLGDEGQKTFDILQGLPHP-
MAQMPLYPVTDIETDYPSWELYGEGLLLDHADVAIFDAACLENSPLPRQHILTSPMLGDNRQVCPSYVVAAELDVLRDEAFAYANQLK
SFGIAVQTHTVLGAPHGFIHFMSVHQRLGQETQHIITGFANFVREIIKTRALLSA

Figure S1. *Psychrobacter* SC65A3.1 *lip2* gene nucleotide sequence cloned in pHAT2 vector and corresponding amino acid sequence of PSL2 lipase. (Underlined sequence): cloning restriction sites *Nco*I (CCATGG) and *Bam*HI (GGATCC) in pHAT2 vector; (Bold) start and stop codons of the *lip*2 gene .

Table S1. Amino acid composition of PSL2.

Amino acid	Number	Content
Ala (A)	40	8.3%
Arg (R)	15	3.1%
Asn (N)	20	4.1%
Asp (D)	32	6.6%
Cys (C)	10	2.1%
Gln (Q)	25	5.2%
Glu (E)	28	5.8%
Gly (G)	29	6.0%
His (H)	21	4.3%
Ile (I)	31	6.4%
Leu (L)	56	11.6%
Lys (K)	23	4.8%
Met (M)	11	2.3%
Phe (F)	19	3.9%
Pro (P)	24	5.0%
Ser (S)	32	6.6%
Thr (T)	28	5.8%
Trp (W)	6	1.2%
Tyr (Y)	11	2.3%
Val (V)	22	4.6%
Pyl (O)	0	0.0%
Sec (U)	0	0.0%
(B)	0	0.0%
(Z)	0	0.0%
(X)	0	0.0%

Total number of negatively charged residues (Asp + Glu): 60
Total number of positively charged residues (Arg + Lys): 38

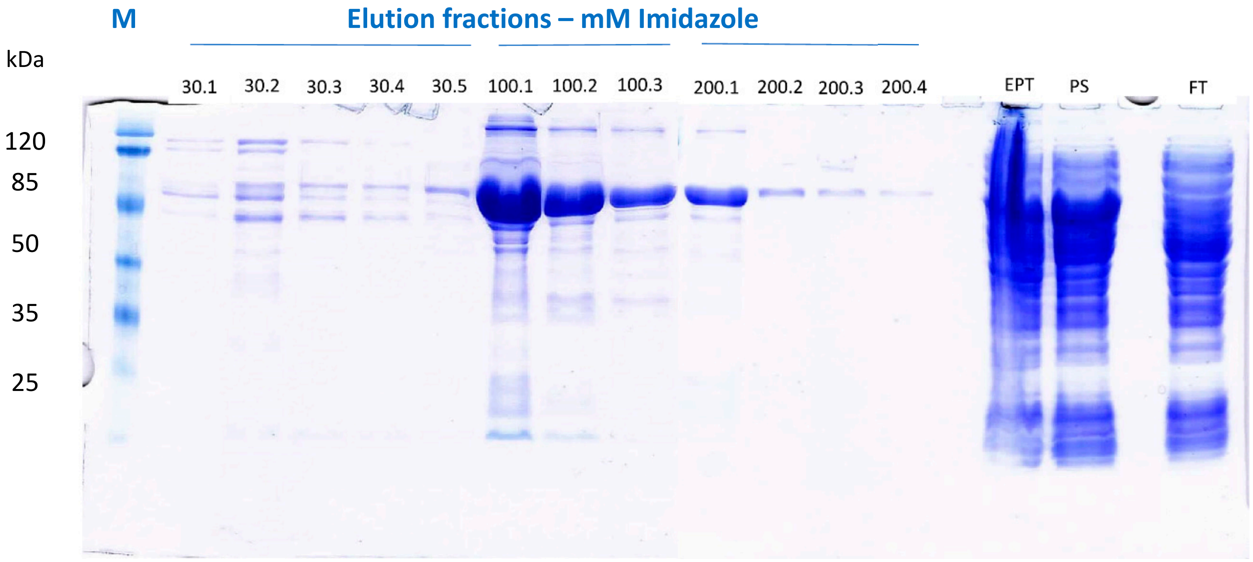

Figure S2. Purification of the recombinant PSL2 by affinity chromatography on Ni-NTA column. The purification fractions analyzed by SDS-PAGE (M): standard molecular mass proteins (Thermo Scientific); (30.1-30.3, 100.1-100.3, 200.1-200.4) fractions containing various concentrations of imidazole (30 mM, 100 mM, 200 mM) in TN buffer (100 mM TrisHCl, pH 8, 200 mM NaCl). (EPS): total extract; (PS): soluble extract; (FT): flow through.
